# Diagnostic Value of Simultaneous Measurement of Procalcitonin, Interleukin-6 and hs-CRP in Prediction of Early-Onset Neonatal Sepsis

**DOI:** 10.4084/MJHID.2012.028

**Published:** 2012-05-06

**Authors:** Alireza Abdollahi, Saeed Shoar, Fatemeh Nayyeri, Mamak Shariat

**Affiliations:** 1 Division of Pathology, Imam Hospital Complex, Tehran University of Medical Sciences Tehran, Iran; 2 Division of Neonatalogy, Imam Hospital Complex, Tehran University of Medical Sciences Tehran, Iran

## Abstract

Neonatal sepsis is a major cause of morbidities and mortalities mostly remarkable in the third world nations.We aimed to assess the value of simultaneous measurement of procalcitonin (PCT) and interleukin-6 (IL-6) in association with high sensitive- C reactive protein(hs-CRP) in prediction of early neonatal sepsis.

A follow-up study was performed on 95 neonates who were below 12 hours (h) of age and had clinical signs of sepsis or maternal risk factors for sepsis. Neonates were assigned to 4 groups including “proven early-onset sepsis”, “clinical early-onset sepsis”, “negative infectious status”, and “uncertain infectious status”. Blood samples were obtained within the first 12 h of birth repeated between 24 hours and 36 hours of age for determination of serum levels of PCT, IL-6, hs-CRP, and white blood cell (WBC) count.

On admission, neonates with sepsis had a higher WBC count, IL-6, PCT, and hs-CRP levels compared with those neonates without sepsis. This remained significant even after 12–24 hours of admission. Also, patients with clinical evidences of sepsis had a higher serum level of PCT and IL-6 within 12–24 hours after admission compared to the patients with uncertain sepsis.

The combination of PCT and IL-6 yielded had a sensitivity of 88% and PCT and CRP (using the cutoff value of 8 mg/L) a sensitivity of 82%.The areas under the ROC curve for the two periods were 0.801, and 0.819 respectively.

In final The combination of IL-6, hs-CRP, and PCT seems to be predictive in diagnosis of early onset neonatal sepsis.

## Introduction

Neonatal sepsis is a major cause of morbidities and mortalities mostly remarkable in the third world nations.^1^–^3^ Early diagnosis and subsequent therapy for the infected infants or those with a higher risk may play a vital role in lowering such mortality and morbidity rates.^4^ Neonatal sepsis has been divided into two main categories including early-onset (within 72 hours of age) and late-onset (after 72 hours of age) sepsis.^1^,^3^,^5^–^7^ Unfortunately signs and symptoms of early-onset neonatal sepsis such as respiratory distress and irritability are vague and not confined to the diseases;^8^ other non- infectious processes also may present with the same manifestations.^4^,^7^,^9^ Conventional methods for diagnosis of sepsis lack enough sensitivity and specificity.^10^ A positive blood culture would be reflective of an asymptomatic underlying bacteremia with less concern while a negative result for blood culture may not assure lack of infection.^11^

The outcome of sepsis in neonates is directly related to the early management of the disease, and hence prompt use of proper antibiotic therapy is absolutely essential.^12^ However, in order to prevent microbial resistance induced by unnecessary administration of empiric therapies and avoiding overwhelmed hospital admissions, a definite diagnosis should be secured based on the laboratory tests with higher diagnostic value.^2^,^9^–^11^,^13^

Procalcitonin (PCT), one of the precursors of calcitonin, is a 116 amino acid peptide. Being produced by an unknown source (it may be produce in the liver or by macrophages), it is secreted into the blood circulation during infection without increasing calcitonin.^14^ PCT is detectable in the plasma as early as 2 h after the exposure to the bacterial products; its level rises for 6 to 8 h, reaches a plateau after 12 h, and then decreases to a normal level after 2 to 3 days.^15^

Recently PCT has received an important role in the diagnosis of bacterial infection in both pediatric and adult population; however, its accuracy in neonatal early-onset sepsis is still an issue of controversy.^14^,^16^ Acute phase reactants and interleukins are also used in the diagnosis of bacterial infection in the neonates. Among these, interleukin-6 (IL-6) is the most well known marker.^17^–^20^

Direct measurement of serum level of cytokines could make the earlier detection of infection possible compared to the measurement of acute-phase reactants which in turn are secreted in response to the proinflammatory cytokines. Hence our study aimed to assess the value of simultaneous measurement of PCT and IL-6 along with the white blood cell, neutrophil and lymphocyte count, blood culture and high sensitive C- reactive protein (hs-CRP) in neonate populations with a rapid diagnosis of the infection.

## Methods

A prospective cross sectional study was conducted among newborns admitted to the neonatal intensive care unit (NICU) of Vali-e-Asr hospital affiliated to Tehran University of Medical Sciences (TUMS) in Tehran, Iran, between January 2009 to January 2010. Newborns were considered eligible if they met the following inclusion criteria: age was below 12 h, they had clinical signs of sepsis, or there was a maternal risk factor for sepsis. Neonates with congenital malformations and TORCH infection were excluded from study.

Clinical signs were considered positive if three or more of the following features were present: 1. respiratory manifestations (Apnea is a term for suspension of external breathing. During apnea there is no movement of the muscles of respiration and the volume of the lungs initially remains unchanged,apnea for at least 10 seconds, tachypnea defined as respiratory rate over 70 / min in preterm and over 60 / min in term neonates), nasal flaring, retractions, cyanosis, or respiratory distress); 2. bradycardia defined as heart rate less than 100 beats / min in preterm and less than 80 beats /min in term neonates or tachycardia (The upper threshold of heart rate based on age :1–2 days:>159 beats per minutes ,bpm, ,3–6 days :>166 bpm,1–3 weeks :>182 bpm,1–2 months :>179 bpm) ; 3. hypotonia or seizures; 4. poor skin color or capillary filling time >2 s; and 5. Irritability or lethargy.

Informed consent was obtained from the parents of the neonates for entering the study and the research and ethic committee of the TUMS approved the study protocol.

### Sampling

Blood samples were obtained by arterial canula within the first 12 h and then repeated between 24 h and 36 h of age. Samples were centrifuged within 30 minutes of collection while the serum stored at −20° C before analysis for determination of PCT, IL-6, CRP. CBC differentiation was measured using Sysmex XT-1800 cell counter, Japan; IL-6 measurement was also performed using a quantitative sandwich enzyme-linked immunoassay technique (Human IL-6 immunoassay, Bioscience, Austria while hs-CRP was assessed by a two-site enzyme-linked immunosorbent assay (Elisa) (biosystem, Barcelona, Spain). PCT measurement was performed using a immunochromatografic test (Brahms, German).

### Infection detection

Based on criteria of Center for Disease Control (CDC) for diagnosis of neonatal sepsis, our patients were selected as following: having postnatal signs of sepsis and a positive blood culture sampled from peripheral or central venous lines (21).

Neonates were assigned to 4 groups including proven early-onset sepsis, clinical early-onset sepsis, negative infectious status, and uncertain infectious status. Neonates with positive blood culture were classified as “proven early-onset sepsis” or “positive blood culture sepsis”. Neonates with negative blood culture, who had positive clinical signs of sepsis (3 or more of 5 aforementioned categories), and positive sepsis screen (based on band cells > 20%, polymorphocytosis, ESR> 55 mm, suggestive CSF analysis, and positive history of chorioamnionitis or PROM) or a maternal risk factors were classified as “clinical early-onset sepsis”. “Negative infectious status group” included neonates with negative blood culture and negative sepsis screen. Neonates with negative blood culture who had only one or no abnormal value of the sepsis screening test and fewer than three clinical signs of infection were classified as “uncertain infectious status”. All the cases proved clinically or by culture to be blood positive underwent intravenous antibiotic therapy. In addition, appropriate care was delivered in neonatal intensive care unit (n-ICU) in case of general condition's instability.

### Statistical Analysis

The statistical package SPSS 17 for windows (Chicago, Illinois, USA), was used for analysis.

After descriptive analysis in the case of non-normal data, the Kruskal–Wallis test was used instead. Variance homogeneity was checked with the Levene test.

The reliability of analytes concentration for the diagnosis of sepsis was calculated by receiver-operating characteristics (ROC) curves. Youden's index (sensitivity + specificity − 1) was used for determination of optimal cutoff values of the diagnostic tests in the different postnatal periods. Sensitivity, specificity, and the likelihood ratio of positive and negative results with a 95% confidence interval (CI) were calculated. Statistical significance was set at *P* < 0.05.Variables are presented as mean ± standard error of mean (SEM). For comparison of serum PCT, IL- 6, and hs-CRP between the groups, one way analysis of variance (ANOVA) were employed followed by post hoc tests.

## Results

Of ninety five neonates who were included in our study, 62 were female (65.3%) and 33 (34.7%) were male. Mean± SD age of the patients was 6.59±0.34 h; mean± SD of weight was 3106.58±65.193 grams among our patients. Cesarean section rate was 61.1% in our study. In addition, 25(26.3%) of mothers were reported to have a fever while 9 (9.4%) of them were diagnosed as preeclampsia. In 6 (6.3%) of the cases, premature rupture of membrane had occurred.

Primary characteristics of the patients along with clinical signs of the sepsis are presented in **table 1**. There were 79 neonates with sepsis i.e. 30 uncertain, 30 positive blood culture, and 19 clinical cases of sepsis, while 16 neonates had no sepsis. Mean age of the neonates in each group did not differ significantly (p> 0.05).

On admission, neonates with sepsis (uncertain sepsis, clinical sepsis, and positive blood culture sepsis) had a significantly higher WBC count, and serum levels of IL-6, PCT, and hs-CRP than those neonates without sepsis (p< 0.01). This remained significant even after 12–24 hours of admission (**Table 2**).

Patients with clinical evidence of sepsis had a significantly higher serum level of IL-6 compared to the patients with uncertain sepsis at the time of admission (p< 0.05). They had a higher serum level of PCT and IL-6 on 12–24 hours after admission when compared to the patients with uncertain sepsis. Patients with positive blood culture sepsis had also higher values of IL-6 compared to the patients with clinical sepsis at the time of admission.

The correlation coefficients between studied variables in the different groups are as follows: serum levels of PCT were significantly correlated with serum levels of IL-6 in patients with no sepsis (r=0.52, p < 0.05) and uncertain sepsis (r=0.34, p<0.05). However, there were not any significant correlation between serum levels of IL-6 and PCT in patients with clinical sepsis (r=0.03, p=0.5) and positive blood culture sepsis (r=0.07, p=0.65). Hs-CRP was significantly correlated with PCT in patients with positive blood culture sepsis (r=0.54, p<0.001), 12–24 hours after admission.

The sensitivity, specificity, positive and negative predictive values of the diagnostic tests within 12 h of life at different cut-off values are listed in **Table 3**.

The combination of PCT and IL-6 yielded a sensitivity of 88%, the combination of PCT and CRP (using the cutoff value of 8 mg/L) a sensitivity of 82%, neither combination being significantly higher than with PCT alone. Sensitivity of PCT (76%) compared with that of CRP (48%) was significantly (*p* = 0.011) different.

We elaborated ROC curves considering infected and non-infected. The areas under the ROC curve for the two periods were 0.801(95% CI, 0.734 to 0.868), and 0.819(95% CI, 0.749 to 0.890), respectively. Cutoff levels with the optimum diagnostic efficiency derived from the ROC curves were ≥ 4.7 ng/mL at 12–24 h of life (sensitivity 72.8%, specificity 80.4%), and ≥ 1.7 ng/mL at 36–48h of life (sensitivity 76.6%, specificity 78.2%).

## Discussion

Procalcitonin is an acute phase protein and in close correlation with IL-6, TNF- α, and CRP and increases remarkably early after inflammation.^22^–^24^ CRP has been used as a diagnostic marker of sepsis for years until IL-6 added to its accuracy by providing a double combination.^22^,^25^ Several studies have showed controversies regarding the ideal marker; Døllner et al. have showed that IL-6 and CRP combination are better diagnostic parameters compared to other inflammatory phase reactants including p55, p75, ICAM-1 and E-selectin.^25^ In nosocomial neonatal sepsis, PCT has received variable accuracy as a diagnostic biomarker. Auriti et al. have reported an increased diagnostic accuracy for PCT in neonates with birth weight lower than 1500 grams;^26^ while López Sastre et al. concluded from one hundred infants that PCT is not sufficiently a sole marker of nosocomial neonatal sepsis and this would be a part of full sepsis evaluation.^13^ Simultaneous measurement of serum markers in diagnosis of early onset neonatal infection have also been suggested by combination of IL-8 and CRP by Frnaz et al.^27^

Our study aimed to determine if procalcitonin could add to the predictive values of other serum biomarkers when approaching to the neonatal sepsis by performing a diagnostic combination for sepsis. The diagnostic benefits of PCT in diagnosis of sepsis have been widely investigated in the studies.^28^,^29^ However, it has been cautioned that this could not serve as a sole marker in detection of neonatal sepsis and should be a part of a full sepsis work up.^13^

The main problem with PCT as a diagnostic tool in neonates is the physiological increase over the first 48 h of life and we must differentiated it from infection.

Our results showed that WBC, IL-6, hs-CRP, and PCT values were higher among neonates with sepsis compared with those without sepsis either at the time of admission or at the end of the first day of life. Sever inflammatory response is what the studies point to as an important cause for increased level of IL-6, hs-CRP, PCT, and WBC.^8^, ^13^–^15^, ^17^–^19^, ^25^, ^29^–^30^ There is no doubt that inflammatory biomarkers are elevated in neonatal sepsis but there are controversies if some of these combinations could play a faster role in an earlier onset phase. WBC has been reported to be of less diagnostic value in neonatal sepsis.^31^ CRP is also believed to elevate during other inflammatory process and does not provide an efficient specificity in neonatal sepsis;^22^ this however does not include PCT and hence it seems that PCT would find its way among diagnostic markers soon either in a solo^22^ or combinative setting.^25^

In our study, IL-6 was higher in patients with clinical evidences of sepsis and even higher in those with a positive blood culture. Evidences confirm that IL-6 levels increase rapidly after exposure to bacterial components.^8^, ^29^, ^31^ A very short half life of IL-6 makes it undetectable in 24 hours after the onset of illness; so a specificity and sensitivity of 89%, 67%, 58 % passes in 0, 24, and 48 hours of neonatal sepsis, respectively.^22^ It seems that while there are infective particles in the blood, IL-6 could be detected in order to secure the diagnosis of sepsis.

In our study, PCT was also higher in clinically proven sepsis patient compared to cases of uncertain sepsis; PCT begins to increase in 2 h of sepsis onset and precedes the increased level of IL-6 and CRP.^15^,^30^ Besides, in younger children, the diagnostic value of PCT even is higher in comparison to other infectious markers such as CRP and IL-6.^28^ In neonates, PCT continues to rise within first 48 hours of life^15^, ^27^, ^29^ and even its level is also impressed by non infectious conditions including asphyxia, hypoxemia, and intracranial hemorrhage.^15^

IL-6 serum level was shown to be correlated directly with levels of PCT in the patients of uncertain sepsis and without sepsis in our population; however, this was not significantly different between another two groups of clinical and positive blood culture sepsis. IL-6 although rises sharply after exposure to the bacterial products, falls rapidly as time elapse and become undetectable in 24 hours.^20^,^21^. In contrast, PCT begins to rise within this duration and this may cause the separation of serum levels of these markers despite the correlation which is seen among individuals without sepsis or of uncertain sepsis.

Our results demonstrate that hs-CRP play a role in the early diagnosis of neonatal sepsis when cut-off values are lowered, according to the results of the ROC analysis.

Our study showed that hs- CRP was correlated with the levels of PCT in positive blood culture sepsis after 12–24 h of admission. In contrast to the narrow diagnostic window of IL-6, CRP is capable to be diagnostic in later time after the initiation of infection or its symptoms.^26^ It seems that both PCT and hs- CRP are late markers which would improve the diagnostic probability in clinical setting. This may be the reason why these two markers are correlated in neonates with proven blood infections in a late time i.e. 12 to 24 hours after admission.

According to the study’s findings, simultaneous measurement of PCT, IL-6 and hs-CRP is more sensitive in diagnosis of neonate infections. Our results are similar to some other studies.^21^,^23^–^5^ Hence, simultaneous measurement of these tests is recommended for infection diagnosis.

The main limitation of our study is its short term duration of follow up both to secure a prognostic estimation and face later clinical and laboratory challenges; however we took advantage of a relatively large sample size and close similarity between groups in most of the potentially confounding variables. In order to reach a consistent similarity between studies’ results, we need a widely acceptable definition for sepsis so we based upon CDC criteria. Besides, prenatology and gestational histories also should be taken into consideration to resolve varieties behind causes of sepsis. Higher rate of cesarean section (CS) in our study (61.1%) in comparison to other societies could be due to the increasing incidence of section within the last years.^22^–^25^ Although in developed countries this has occurred with a slower pace, in low income nations, it could be experienced by a much more ascending steep.^22^ Also in our country of Iran, the overall rate of CS has been showed to be 85.3% 22 of which most occurs as a result of repeated section. This however was not associated with an interfering effect as the rate of section did not differ significantly between the four assigned groups. In order to define the most proper serum marker to diagnose neonatal sepsis on time, one should obtain sufficient sensitivity and specificity; besides, it should be detectable based on the defined cut offs either in an early phase or at the later time. Considering that no single marker seems to provide all the mentioned characteristics, so far all the recommendations are pointing to a combination of inflammatory markers as a part of diagnostic work up for neonatal sepsis. These combinative diagnostic approaches should receive more evidence to be widely applicable in clinical practice.

## Conclusion

We showed a higher serum level of PCT, hs-CRP, and IL-6 in neonates with sepsis compared to those without sepsis. However, due to the lack of consensus in definition of sepsis and because of the heterogeneity in the statistics of our studies just as in the others’ studies, attention should be paid in interpreting the results. This study paves the road for future studies in determining sensitivity and specificity of diagnostic tests in early phases of neonatal sepsis.

## Figures and Tables

**Table 1:** The primary characteristics of the participants

**Age(hours)**		6.59±0.34
**Weight(g)**		3106.58±65.193
**Head circumference (cm)**		34.96±0.136
**Apgar Score on Admission**	**5**	5(5.3%)
	**6**	9(9.5%)
	**7**	13(13.7%)
	**8**	44(46.3%)
	**9**	24(25.3%)
**Route of Delivery**	**Cesarean Section**	58(61.1%)
	**Vaginal Delivery**	37(38.9%)
**Gestational age**	**32**	6(6.3%)
	**33**	6(6.3%)
	**34**	5(5.3%)
	**35**	18(18.9%)
	**36**	18(18.9%)
	**37**	30(3.6%)
	**38**	12(12.6%)
**Apnea**		6(6.3%)
**Tachypnea**		23(24.2%)
**Tachycardia**		18(18.9%)
**Hypotonia**		35(36.8%)
**Seizure**		5 (5.3%)
**Lethargy**		20(21.2%)
**Hypersensitivity**		29(30.5%)
**Nasal Flaring**		8(8.4%)
**Poor Skin Color**		12(12.6%)
**Premature rupture of the membrane**		6(6.3%)
**Cyanosis**		8(8.4%)
**Distress**		47(49.5%)
**Bradycardia**		2(2.1%)
**Capillary Filling**		25(26.3%)
**Jaundice**		25(26.3%)
**Maternal Fever**		25(26.3%)
**Preeclampsia**		9 (9.4%)

**Table 2.** Comparing the data of the patients with uncertain sepsis, clinical sepsis, blood culture positive sepsis and controls.

		Without Sepsis (n=16)	Uncertain sepsis (n=30)	Clinical Sepsis (n=19)	Blood Culture Positive Sepsis (n=30)	P value
**Age (hours)**		7.25±0.93	6.2±0.37	6.32±0.78	6.22±0.58	NS
	**White blood cell Count (× 109/l)**	8.40±1.00	16.40±1.00	15.25±0.731	17.343±1.51	<0.001
**On admission**	**Lymphocyte %**	29.1±1.58	29.1±1.58	28.8±1.89	28.8±1.74	NS
	**Neutrophil %**	54.8±3.13	61.8±3.13	60.0±2.19	62.6±1.77	NS
	**Hs-CRP (mg/L)**	0.887±0.117	1.450±0.25	2.73±0.507	2.9±0.50	<0.001
	**lnterleukin-6 (pg/ml)**	36.75±3.67	43.8±3.07	63.7±3.05	63.7±6.05	<0.001
	**PCT (ng/ml)**	0.57±0.14	1.4±0.35	1.84±0.72	4.3±0.82**	<0.001
	**White blood cell Count (× 109/l)**	8.60±0.37	12.60±0.378	13.20±0/73	10.46±0.793	<0.01
**between. 12 and 24 hours after admission**	**Lymphocyte %**	31.0±2.94	38.0±2.94	38.16±2.60	39.3±2.61	<0.05
	**Neutrophil %**	53.75±2.25	55.75±2.25	55.41±2.25	52.57±1.72	NS
	**Hs-CRP (mg/L)**	2.22±0.27	3.22±1.02	4.61±1.35	6.21±1.05	<0.001
	**lnterleukin-6 (pg/ml)**	45.9±3.65	75.5±5.99**	117.5±9.39	120.6±8.31**	<0.001
	**PCT (ng/ml)**	0.55±0.239	1.7±0.23*	3.14±1.69	4.2±2.5	<0.001

Variables are expressed as Mean ± Standard error of mean (SEM). Variable are compared using student t test.

*: P<0.05,

**: P<0.01, when comparing neonates with uncertain sepsis vs. neonates with clinical sepsis and comparing neonates with uncertain sepsis vs. neonates with blood culture positive sepsis. The column “p value” is presented, when comparing neonates with blood culture positive sepsis with controls. Hs-CRP, high sensitive- C reactive protein, PCT: procalcitonin; NS: not significant.

**Table 3:** Sensitivity, specificity, positive (PPV) and negative predictive value (NPV) of 12-h values of procalcitonin, interleukin-6 and hs-CRP,determined by ROC analysis using the Youden’s index for optimal cut-off values in neonates with blood-culture-positive and clinical sepsis compared to neonates with negative infectious status.

**Diagnostic test**	**Cut-off**	**Sensitivity (%)**	**Specificity (%)**	**PPV (%)**	**NPV (%)**	**Youden**
**Procalcitonin(ng/ml)**	≥1.7	76.6	78.2	93	72	0.684
	≥4.7	72	80.4	76	70	0.437
**Interleukin-6(pg/ml)**	≥60	54	100	100	59	0.543
	≥10	71	65	76	60	0.366
	≥150	46	100	100	55	0.457
**hs-C-reactive protein(mg/L)**	≥2.5	69	96	96	67	0.642
	≥8	49	100	100	58	0.486
